# Nurses’ Challenges in Performing Ultrasound Hip‐Screening Techniques for Home Visits to Newborns and Infants: A Descriptive Case Study

**DOI:** 10.1155/jonm/8226375

**Published:** 2026-04-13

**Authors:** Mana Shirouchi, Hiroshige Matsumoto, Chikako Honda, Misa Shiomi, Noriko Hosoya, Takeshi Kinjo, Kiyoshi Aoki, Tadashi Hattori, Keita Okada, Kenta Fujiwara, Kyoko Yoshioka-Maeda

**Affiliations:** ^1^ Department of Community Health Nursing/Public Health Nursing, Division of Health Sciences and Nursing, Graduate School of Medicine, The University of Tokyo, 7-3-1 Hongo Bunkyo-ku, Tokyo, Japan, u-tokyo.ac.jp; ^2^ Innovative Public Health Nursing, Graduate School of Medicine, Kyoto University, 53 Shogoin-Kawara-cho Sakyo-ku, Kyoto, Japan, kyoto-u.ac.jp; ^3^ Department of Nursing, Faculty of Healthcare Sciences, Chiba Prefectural University of Health Sciences, 2-10-1 Wakaba Mihama-ku, Chiba-City, Chiba, Japan; ^4^ Okinawa Prefectural Nanbu Medical Center and Children’s Medical Center, 118-1 Arakawa Haebaru Town Shimajiri-Gun Okinawa, Japan; ^5^ Asahigawasou Rehabilitation and Medical Center, 866 Gion Kita-ku, Okayama City, Okayama, Japan; ^6^ Aozora Family Clinic, Pediatric Orthopedic Center, 60-1 Hannoki Yoshida-cho, Obu-City, Aichi, Japan; ^7^ Department of Orthopaedic Surgery, Graduate School of Medicine, The University of Tokyo, 7-3-1 Hongo Bunkyo-ku, Tokyo, Japan, u-tokyo.ac.jp; ^8^ Doi Orthopedic Clinic, 15-8 Takatsuki-machi, Takatsuki, Osaka, Japan

## Abstract

**Aims:**

Early detection of developmental dysplasia of the hip in newborns and infants is essential to ensure lifelong mobility. As several municipalities conduct nurse‐led hip ultrasound screenings during newborn and infant home visits, nurses must acquire the accurate technique smoothly and effectively. Clarifying the challenges nurses face when performing ultrasound procedures helps support effective education. This study aimed to identify challenges inexperienced nurses face when performing basic ultrasound using the Graf‐method in home settings.

**Design:**

Descriptive case study.

**Methods:**

Ultrasound training was provided to 22 nurses in Japan to enable use during newborn and infant home visits. We analyzed 25 video cases from three nurses who required > 180 s to obtain a standard plane image. Videos were transcribed and inductively analyzed to identify challenges with reference to Yin’s case study methodology.

**Results:**

Conditions of the challenges were categorized into knowledge, skills, and attitudes with reference to Bloom’s taxonomy. The knowledge gap led to the failure to correctly judge whether the femoral head/standard plane was captured. Skill deficiencies led to failure to move the probe vertically and difficulty maintaining the infant’s straight lateral position. The combination of inadequate knowledge and skills resulted in failures to (1) place the probe directly over the femoral head, (2) display the image in the correct position and orientation, and (3) move the probe correctly regarding direction and range while maintaining the image in the correct position and orientation. Regarding attitude gaps, nurses failed to improve the procedure.

**Conclusions:**

Improving ultrasound proficiency for developmental hip dysplasia detection requires training in anatomy integration, sensory experience, and probe‐screen coordination while addressing posture, equipment handling, and motor skills.

**Trial Registration:** University Hospital Medical Information Network Clinical Trial Registry: UMIN000051929

## 1. Introduction

Delayed diagnosis of developmental dysplasia of the hip (DDH) significantly affects children’s lifelong mobility [1], often leading to osteoarthritis and hip replacement surgery in adulthood [2]. The prevalence of DDH among infants is approximately one in 100, with a slight upward trend in the overall lifetime DDH prevalence [3]. Although physical examination remains the initial method for detecting DDH, its 50.5% sensitivity and 75.0% specificity may cause uncertainty and delayed diagnosis [4]. Although early detection of DDH is vital for improving outcomes and reducing costs [1, 5], promoting accurate screening remains challenging.

## 2. Background

Universal ultrasound screening is a widely recommended method that enables early DDH detection, improves detection rates, and reduces surgery rates, complications, and costs [5–8]. The Graf‐method is a globally used, fast, and standardized ultrasound examination method that enables precise morphological assessment of hip joint components, allowing for the early identification of potential DDH [8–10]. Physicians frequently use this method because of its high reliability and the availability of assistive tools such as foot switches, probe holders, and Graf’s fixation device in clinical settings [10–12]. These tools help maintain probe positioning vertically and improve the diagnostic accuracy of hip dysplasia in infants [10, 11, 13]. The practitioner applies an ultrasound probe over the femoral head of the infant’s hip joint, capturing the standard plane (SP) of the iliac crest, inferior border of the ilium, and acetabular labrum to assess the hip joint’s condition [10]. One challenge of ultrasound screening is the limited availability of physicians and hospitals, which makes timely DDH screening difficult for rural residents and exacerbates health disparities. In Japan, public funding supports a system in which nurses visit the homes of all newborns and infants [14, 15]. With appropriate training, nurses have demonstrated diagnostic accuracy comparable to that of junior physicians in clinical settings [16]. Furthermore, high‐quality images can be obtained without Graf’s fixation device, provided the infant is securely stabilized by hand [12]. Therefore, home ultrasounds for newborns and infants, performed by nurses, represent a feasible and effective outreach strategy to reduce health disparities in medically underserved areas.

The ultrasound examination for DDH requires a precise technique to capture the SP. Prior studies have partially clarified the technical difficulties encountered by physicians and nurses during the imaging phase of ultrasound examinations in clinical settings [16, 17]. When training is insufficient, even physicians tend to require longer examination times and experience reduced reproducibility, including deviations in the inclination of the iliac outer margin relative to the vertical axis, under the Graf‐method [17]. Junior physicians and nurses with no prior ultrasound experience often make errors, such as missing lower limbs, using incorrect measurement lines, and encountering technical issues, including probe angulation, in a clinical setting [16]. Inaccurate image acquisition caused by probe angulation can lead to misdiagnosis [13]. A previous study found that nurses face limited training opportunities and a shortage of mentors for ultrasound examinations, including point of care ultrasound, resulting in insufficient image‐acquisition skills and lower confidence [18]. Proficiency levels vary among nursing professionals participating in ultrasound training programs [19]. Past reports indicate that approximately 38%–97% of nursing participants succeed in acquiring ultrasound skills [20, 21]. Therefore, analyzing errors to gain insights and providing educational support for nurse trainers are essential steps toward advancing from basic physical examinations to high‐quality, safe ultrasound procedures [22].

Recently, standardized procedures and educational programs have been developed to enhance nurse‐led hip screening using the Graf‐method ultrasound during home visits for early detection of DDH [23, 24]. Home‐based ultrasound examinations differ from those in well‐equipped clinical settings with assistive tools. Transporting medical equipment to patients’ homes places substantial burdens on healthcare providers and often conflicts with the constraints of individual home environments. Consequently, (1) the probe may need to be held manually with both hands when a holder is unavailable and (2) when positioning pillows are unavailable, an inexperienced caregiver may need to stabilize the infant manually [12]. Therefore, home settings may involve greater practical challenges and reduced image stability—particularly regarding probe control and infant stabilization—compared with clinical environments. However, previous studies have been primarily clinical‐based, and the technical difficulties faced by nurses performing DDH ultrasound examinations in home settings remain unclear. Addressing this gap requires investigating the challenges nurses face when acquiring and applying ultrasound knowledge, skills, and attitudes during training. Understanding these challenges could lead to the development of more effective educational programs and improve nurses’ performance in home‐based DDH screening.

## 3. Study Aim

This study aimed to describe the challenges nurses encounter while learning and performing ultrasound hip screening using the Graf‐method during an educational program focused on home visits. The research question was as follows: How and why do nurses with limited experience performing basic ultrasound examinations using the Graf‐method struggle to capture SP during ultrasound training in home settings?

## 4. Methods

### 4.1. Design

We conducted a descriptive case study based on video observations. The analytical procedure was conducted with reference to Yin’s case study methodology [25]. A case study design is suitable for exploring questions related to a phenomenon’s method, content, and underlying causes [25]. We determined that this design was suitable because we aimed to retrospectively examine instances in which inexperienced nurses conducted ultrasound examinations and to identify the patterns and reasons behind potential challenges. We followed the SRQR guideline for reporting qualitative research [26].

### 4.2. Theoretical Framework

We referenced Bloom’s taxonomy [27–29]. Bloom categorized learning outcomes into three domains: cognitive (knowledge), psychomotor (skills), and affective (attitudes) [27, 29]. The cognitive domain involves acquiring information through learning and is related to understanding and knowledge. The psychomotor domain involves acquiring motor skills to perform specific tasks and is related to physical actions. The affective domain involves reactions to new information and relates to emotions and attitudes. These three domains are essential for effective education [27, 29]. This theoretical framework provides a perspective for developing and assessing nurses’ clinical competencies, thus enabling the identification of learning gaps and weaknesses [30]. This study focused on providing an ultrasound education program and addressing the challenges nurses face when acquiring knowledge, skills, and attitudes. Therefore, Bloom’s taxonomy was considered an appropriate theoretical framework for a good understanding of the phenomena targeted in this study. This study aimed to improve the knowledge, skills, and attitudes within an educational program.

### 4.3. Study Setting and Recruitment

This study was conducted in three municipalities in Japan. We conducted an educational program for nurses using a pre–post design as part of a research project to prevent overlooked patients with DDH in community settings for newborns and infants [23]. This program was developed based on discussions with medical experts who supervised hip ultrasound screening in hands‐on seminars conducted by the Japanese Society of Orthopedic Ultrasonics (T.K., K.F., and K.A.). The program consisted of three components: (1) e‐learning (93 min), (2) hands‐on seminars with Objective Structured Clinical Examination (OSCE) (105 min), and (3) clinical training with infant volunteers in simulated home settings (120 min) [23, 24]. The e‐learning component covered ultrasound basics, anatomy of the hip joint (with two‐ or three‐dimensional anatomical illustrations), principles of the Graf‐method and ultrasound image interpretation, practice of the Graf‐method (including key points of probe manipulation), and ultrasound practice and health education in newborn home visits [23, 24]. Each e‐learning module included a mini‐test, and participants progressed only after passing. OSCE criteria adhered to previously validated standards: participants were evaluated across 36 items covering preparation (8 items), procedure (18 items), SP image acquisition (4 items), caregiver education (4 items), and image description and interpretation (2 items) [19]. Each item was scored as 0 (unable to perform despite advice), 5 (able to perform with advice), or 10 (able to perform without advice), resulting in a total possible score of 0–360 points [19]. During the OSCE, the time required for nurses to acquire an SP image was measured separately for each hip of the phantom, recorded in seconds.

### 4.4. Inclusion and Exclusion Criteria

**FIGURE 1:**
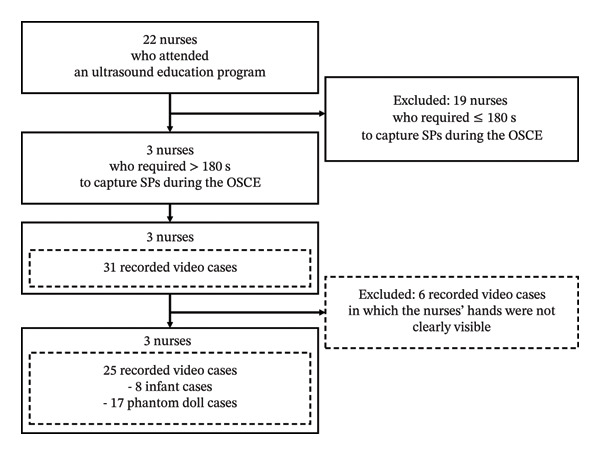
Study flowchart.

Of the 22 nurses who participated in this program, this study included three who required > 180 s to capture the SP during the OSCE (Figure 1). The exclusion criterion was nurses who captured SP in ≤ 180 s. The time threshold was set at 180 s based on two considerations: the maximum recording duration per session of the ultrasound device (iViz air Ver.5 linear, Fujifilm, Tokyo, Japan) and the observation that this duration exceeds one standard deviation above the mean scan time recorded for 22 nurses (right hip: 89.21 s, SD = 66.58 s; left hip: 81.58 s, SD = 74.03 s). This threshold was used as a pragmatic criterion to identify cases potentially indicative of insufficient learning outcomes among the 22 nurses [27, 29].

The inclusion criterion for videos from the three participants was clear visibility of the nurses’ hands, recorded between the hands‐on seminar and clinical training; the exclusion criterion was any lack of visibility. When other nurses observed the ultrasound examination or when the authors provided guidance, the nurses’ hands were sometimes obscured and not captured on camera, resulting in the exclusion of six cases.

### 4.5. Devices

An ultrasound device, iViz air Ver. 5 linear (Fujifilm, Tokyo, Japan), was used during this program. The probe dimensions were 57.9 (width) × 170.5 (height) × 29.0 mm (depth), with a weight of 147 g. The battery was built into a handle. The phantom doll was a baby‐shaped device from Kyoto Kagaku (Kyoto Kagaku Co., Ltd., Kyoto, Japan) that allows visualization of the hip joint when an ultrasound device is applied.

### 4.6. Data Collection

All the nurses and caregivers of the infant volunteers who underwent ultrasound examinations provided informed consent to participate in the study and completed a questionnaire on their basic characteristics.

Second, nurses performed ultrasound examinations in a simulated home setting using a phantom doll in the OSCE and both a phantom doll and infant volunteers in clinical practice. We recorded videos of nurses, phantom dolls, infant volunteers, and caregivers participating in ultrasound education programs based on the Graf‐method held at public facilities in each municipality [23]. The authors recreated a simulated home environment in a room within a public facility. For safety reasons, the examination was standardized to be conducted on the floor rather than in a crib, with the caregiver providing support for the infant’s body. The minimum required space was sufficient for both the caregiver and the nurse to sit on the floor, place rolled bath towels along the ventral and dorsal sides of the infant in a side‐lying position, and position a tablet device immediately beside the infant. Although Japanese household environments vary widely, national housing statistics report an average floor space per dwelling of 92 m^2^ [31], indicating that the recreated environment falls within a realistic residential range. Adequate lighting and visibility were ensured through a combination of natural daylight and ceiling lighting. Caregivers sat beside their infant and followed standardized instructions to place a rolled towel in front of their knees to maintain the infant’s alignment by positioning their hands on the infant’s shoulder and knees. Nurses received standardized training through e‐learning and hands‐on seminars to ensure they could explain this fixation method to caregivers and guide its implementation; their competency was assessed through OSCE. Caregivers were provided with prior explanations of the fixation method via a standardized instruction sheet and agreed to follow the nurse’s directions. Pediatric orthopedists pre‐diagnosed the infant volunteers whose videos were used in this study as Type I (normal). Recordings were made during both OSCE assessments and practice sessions. The video camera was positioned 1–3 m away from the nurse to ensure the comfort and privacy of the nurse, infant volunteers, and caregivers. The camera was mainly adjusted to capture the hands and body of the nurse during the ultrasound examination, as well as the phantom doll or body of the infant volunteer. Cameras were set up before the ultrasound education sessions, and fine adjustments were made to ensure that the nurses and phantom dolls/infant volunteers were visible. However, when other nurses observed the ultrasound examination or the authors advised the nurses, the primary nurse was no longer visible on the camera. To prioritize educational opportunities, we refrained from moving the camera between nurses. We considered the need to triangulate the data sources; however, we opted to use only video recordings in this study, considering the burden on nurses, infant volunteers, and caregivers.

### 4.7. Data Analysis

An inductive approach explored the patterns that clarified these concepts with reference to Yin’s case study methodology [25].

First, the first author repeatedly reviewed the video recordings and transcribed the action and spoken content into text. A case was defined as the period from when a nurse began the ultrasound procedure until it ended for any reason. The start time for each case was recorded when the nurse began preparing the ultrasound device, and the end time was defined as either when they successfully captured the SP or reached a time limit of 180 s. If authors asked the nurses about their practice immediately after the end time (e.g., “What did you look at to determine that you captured the SP?”), the end time was recorded as the moment the nurse finished responding. The research questions guided the transcription process, including prompts such as “Who is in the video?” “Where are the nurses, infant volunteers, phantom dolls, and caregivers positioned?” and “What/When/Why are they doing?” This transcription process resulted in 27,773 Japanese characters.

Second, during the coding stage, the first author segmented the text into meaningful units and recorded codes for difficulty and its corresponding condition using Microsoft Excel. A meaningful unit for difficulty defined as an event that hindered or interrupted the procedure leading to SP acquisition. The coding framework was developed based on the literature on the Graf‐method [10, 32] and previous discussions among the authors. The steps are as follows: (1) infant placed in the lateral position; (2) palpate the greater trochanter; (3) place the probe vertically over the femoral head; (4) move the probe back and forth parallel over the greater trochanter to locate the joint; (5) move the probe slightly to locate the lower limb of ilium; (6) rotate the probe to straighten the lower limb of the ilium and obtain the SP; and (7) capturing SP. For example, based on steps (3)–(6), the importance of probe manipulation for successful SP acquisition provided a basis for examining the transcripts. The angle, position, and movement of the probe, as described in the nurses’ actions, were documented and coded as manifestations of difficulty. Meaningful units for condition represented the underlying factors contributing to each difficulty. Bloom’s taxonomy [27–29] was referenced due to its suitability for characterizing factors arising from nurse learning. For example, for difficulties related to probe manipulation, descriptions indicating factors that could influence probe handling—such as finger pressure, hand placement, and wrist stability—were coded. When multiple interpretations or ambiguity arose regarding a difficulty or condition, the first author identified these instances, and coauthors collaboratively reviewed the video recordings and transcripts to reach consensus on the coding. The first author, who conducted the primary analysis, and coauthors (H.M., C.H., K.Y.‐M.) received direct training in DDH ultrasound from a pediatric orthopedic surgeon or Professor. Graf.

Third, within‐case analyses were conducted to examine each case individually and identify patterns using the coded data. This was followed by cross‐case analyses to compare cases, organize recurring patterns and differences, and extract overarching themes [25]. First, codes related to a specific difficulty were compared across similar or distinct situations to evaluate the success or failure of SP acquisition. For example, comparing scenes in which the probe was held vertically with those in which it was angled revealed clear differences in SP acquisition outcomes; probe verticality emerged as a recurring pattern across multiple cases and was considered a candidate theme. Next, conditions associated with each difficulty were examined. For instance, a preliminary hypothesis proposed that pressure applied to the probe might affect verticality. Uniform vertical pressure, however, did not compromise verticality or impede SP acquisition, leading to rejection of this hypothesis. In contrast, uneven pressure consistently corresponded with loss of verticality and subsequent SP‐acquisition failure, supporting inclusion of the code related to “apply pressure unevenly to the probe” in the theme. Through a systematic comparison of each condition relative to its corresponding difficulty across cases, comprehensive themes explaining the success and failure of SP acquisition were generated.

### 4.8. Ethical Considerations

This study was approved by the Research Ethics Committee of the University of Tokyo School of Medicine (Approval No.: 2023101NI). Consent to participate was obtained from all nurses and infant volunteers (with caregivers acting on their behalf). Before the ultrasound, caregivers were informed about the purpose, procedure, expected discomfort, risks, and their right to stop the examination at any time. The infant volunteer’s discomfort was closely monitored throughout the procedure, and the caregiver held the infant’s body at all times alongside the nurse. Only the areas necessary for DDH imaging were exposed, and the infant was promptly redressed with a diaper and clothing under the caregiver’s supervision immediately after the examination. We ensured that the faces of the nurses, infant volunteers, and caregivers were not displayed in the video. The examination was quick and noninvasive, and if the infant cried, breaks or termination of the procedure were implemented as necessary.

### 4.9. Rigor

To enhance conceptual validity and reliability [25], the first author, a public health nurse (PHN) trained in the Graf‐method and with experience in qualitative research, created a verbatim transcript from the video recordings of each case. Three coauthors with similar qualifications reviewed the videos and transcripts to ensure completeness and quality and reached a consensus. Additionally, to improve the consistency of the procedures and analyses, a manual and codebook explaining codes, subthemes, and themes were shared among the authors.

To ensure validity [25], we conducted member checking with the three nurses who participated in the ultrasound training program. These nurses, who did not require > 180 s to capture SP in the OSCE, were familiar with the challenges faced by their peers and worked together on discussions and practices for improvement. This made them well suited to represent the nurses’ experiences. The final categories and subcategories were determined by consensus after member checking and a thorough discussion with the coauthors.

## 5. Findings

### 5.1. Demographics of the Nurse Participants

Table 1 presents the demographic data of the 22 nurses who participated in the program. The three nurses included in this study were in their 20s to 60s. All the nurses were PHNs with no prior experience with DDH screening. Nursing education varied: one had a university degree, one attended vocational school, and one trained at a training school. In addition, three nurses’ e‐learning scores ranged from 43.2 to 49.6 (maximum score 50; no limit on the number of attempts), and total OSCE scores ranged from 272.5 to 313.3 (maximum score 360; only one attempt allowed).

**TABLE 1:** Characteristics of the nurses who participated in the program (*n* = 22).

**Categories**		***n* (%)**

Age	20–29	2 (9.1)^∗∗^
	30–39	3 (13.6)
	40–49	11 (50.0)
	50–59	4 (18.2)^∗∗^
	60–69	2 (9.1)^∗∗^

Certification for infant home visit	Public Health Nurse	19 (86.4)^∗∗^
	Midwife	2 (9.1)
	Registered Nurse	1 (4.5)

Educational Background	Advanced Program at a University	1 (4.5)
	University	10 (45.5)^∗∗^
	Advanced Course at a Junior College	2 (9.1)
	Vocational School	8 (36.4)^∗∗^
	Training School	1 (4.5)^∗∗^

Previous training related to DDH^∗^ screening	Experienced	4 (18.2)
	No experience	18 (81.8)^∗∗^

^∗^DDH: developmental dysplasia of the hip.

^∗∗^Items corresponding to the three nurses included in this study.

### 5.2. Characteristics of the Video Cases

Three nurses conducted ultrasound examinations in 31 recorded videos during an OSCE using a phantom doll and practiced with both infant volunteers and a phantom doll program between December 2023 and August 2024 (Figure 1). Of these, 25 video cases (including 8 infant cases and 17 phantom doll cases) were selected for analysis, excluding 6 video cases where the nurses’ hands were not clearly visible. The total recording time for the video cases selected for the analysis was 3877 s.

### 5.3. Demographics of the Infant Volunteers

The characteristics of the infants who underwent ultrasound examinations by three nurses are shown in Table 2. Four of eight infants were female (50.0%), with a mean age of 130 days (SD = 41.9). Three (37.5%) were firstborn infants. Two (25.0%) had limited hip abduction as recognized by caregivers, while another two (25.0%) had asymmetry of the groin skin folds. None (100%) were aware of a family history of DDH.

**TABLE 2:** Characteristics of the infants who underwent ultrasound examination by the nurses (*n* = 8).

**Categories**		***n* (%) or mean (SD)**

Sex	Male	4 (50.0)
	Female	4 (50.0)

Age in days		130 (41.9)

Birth order	First	3 (37.5)
	Second	3 (37.5)
	Third and beyond	2 (25.0)

Fetal position at birth	Breech position	0 (0.0)
	Others	8 (100.0)

Limited hip abduction as recognized by caregivers	Recognized	2 (25.0)
	Not recognized	4 (50.0)
	Unknown	2 (25.0)

Asymmetry of the groin skin folds as recognized by caregivers	Recognized	2 (25.0)
	Not recognized	5 (62.5)
	Unknown	1 (12.5)

Family history of DDH^∗^	Present	0 (0.0)
	Absent	7 (87.5)
	Unknown	1 (12.5)

^∗^DDH: developmental dysplasia of the hip.

A pediatric orthopedist prediagnosed the infant volunteers whose videos were used in this study as type I (normal).

### 5.4. Challenges Faced by Nurses in Learning Basic Ultrasound Techniques

**FIGURE 2:**
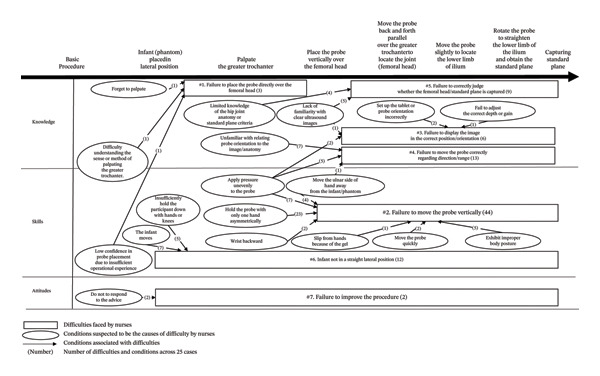
Challenges faced by nurses in learning basic ultrasound techniques.

Nurses learning about DDH and ultrasound for the first time encountered seven challenges in capturing SP (Figure 2). The vertical axis represents knowledge, skills, and attitudes [27–29], whereas the horizontal axis represents basic ultrasound procedures based on the Graf‐method in our educational program [10, 23]. The difficulties faced by nurses are enclosed in squares and positioned along the horizontal axis. The conditions suspected to contribute to these difficulties are enclosed in circles and positioned on the vertical axis. Difficulties related to specific procedural steps are numbered 1–5, whereas those affecting the entire procedure are numbered 6 and 7.

#### 5.4.1. Failure to Place the Probe Directly Over the Femoral Head

Nurses needed to palpate the greater trochanter to position the probe over the femoral head. However, one nurse who did not fully understand the tactile requirements for palpation touched the area only lightly and superficially. Another nurse forgot the necessity of palpation and omitted it altogether, making it difficult to place the probe correctly over the femoral head. Additionally, one nurse had insufficient operational experience and lacked confidence in probe placement, which prevented initiation of palpation. Repeated emphasis on and practice in palpating the greater trochanter and placing the probe helped less confident nurses improve accuracy.

The author asked ID2, “Where will you place the probe?” ID2 responded, “The… greater…? No, not that.” The author repeated, “Greater trochanter.” ID2 replied, “Greater trochanter, got it. […]” while palpating the greater trochanter and placing the probe on the right side of the phantom doll’s hip. (ID2)

#### 5.4.2. Failure to Move the Probe Vertically

Nurses were required to hold the probe vertically above the femoral head to direct the ultrasound beam straight onto the participants’ hips so that the SP was clearly visible. However, three factors made it difficult to maintain this position. First, the probe handling was incorrect. A few nurses applied pressure unevenly on the probe or moved the ulnar side of their hand away from the participant, causing the probe to tilt sideways. Others held the probe with only one hand asymmetrically. A few nurses extended their wrists backward, and the probe slipped from their hands because of the gel. In these videos, the probe was more likely to tilt in multiple directions than when both hands symmetrically held the bottom of the probe. These factors were observed particularly among nurses in their 50s and 60s.

ID1 removed the left hand from the probe, held it with the right hand, and rotated it 5° at once. As ID1 said, “The femoral head is visible…so…,” the probe tilted back and forth, causing the femoral head to be unclear. (ID1)

Second, the probe quickly moved back and forth in an unsteady manner. One nurse rotated the probe so quickly that it became difficult to hold it steady with both hands, which caused the probe to tilt sideways. Especially when the ulnar sides of both hands were not in contact with the participant, the probe shifted away. Third, a few nurses exhibited improper body posture. One nurse focused on the screen displaying the ultrasound image instead of probe positioning, causing her upper body to lean and the probe to tilt and shift over the femoral head.

ID3, kneeling with the upper body tilted forward by about 45°, started peering at the monitor placed to the left, causing the probe in the hand to shift to the left side (phantom doll’s leg side). (ID3)

However, a nurse who objectively assessed the verticality of the probe by checking its position adjusted it correctly and displayed the image.

#### 5.4.3. Failure to Display the Image in the Correct Position/Orientation

Nurses attempting to capture the femoral head or other areas faced four problems with the ultrasound images on the tablet.

First, a few nurses set up the tablet or probe orientation incorrectly, which caused the ultrasound image to appear flipped, making it challenging to accurately identify the anatomical relationships of the hip joint.

ID1 lifted the probe placed on the phantom doll and tilted her head while touching the greater trochanter with her fingers. The pediatric orthopedist (author) placed his right hand on the top of her probe and said, “Is the probe not reversed?” (ID1)

Second, one nurse failed to adjust the correct depth and gain according to the situation. This nurse set the depth to 5 cm while scanning the phantom doll. The image quality was poor due to excessive depth, but the nurse was unsure of the reason and showed signs of confusion.

ID2 said, “The tablet setup: set this here (depth at 5 cm). Gain is in the middle. F mark is down […].” The author, looking at the tablet, advised, “Let us set it to 4 cm; 5 cm makes it a bit difficult, so we will use 4 cm.” (ID2).

Third, when nurses applied pressure on the probe with their hands, it caused it to tilt unintentionally and distort the image.

Fourth, one nurse was unfamiliar with interpreting clear ultrasound images and failed to notice that the image was inverted.

#### 5.4.4. Failure to Move the Probe Correctly Regarding Direction/Range

Nurses were required to slide, rotate, or tilt the probe in different directions to center the femoral head on the screen or display the SP. However, they moved the probe in the wrong direction due to unfamiliarity with relating probe movements to the resulting image and underlying anatomy. For example, one nurse rotated the probe instead of sliding it forward or sideways to center the femoral head, causing it to disappear from the screen.

The author said to ID3, “That looks good. Move the femoral head lower.” ID3 rotated the probe slightly to the left and right about four times. […] The author then held the middle of her probe, adjusted it to a vertical position, moved it back and forth, and said, “Here, the femoral head comes into view.” (ID3)

A few nurses knew the correct probe direction but failed to properly anchor their hands. Applying pressure to the probe with their hands or lifting its ulnar side away from the participant caused the probe to become misaligned or restricted its range of motion.

ID3 applied pressure to the probe with her hand, causing it to press into the infant’s buttocks. She tried to move the probe forward, backward, left, and right by pushing with her thumb, but instead, the probe simply rocked in those directions. (ID3)

#### 5.4.5. Failure to Correctly Judge Whether the Femoral Head/SP Is Captured

Nurses were required to determine whether the femoral head and SP points were visible after positioning and operating the probe above the femoral head. However, one nurse lacked familiarity with the clear ultrasound images of the femoral head and SP, as well as their ideal representation. Consequently, she missed the correct timing for subsequent probe operations and failed to capture the SP.

Although the femoral head was not clearly visible, ID1 said, “I can see the lower limb of the ilium, so I will align the mid‐portion of the bony roof parallel.” At the same time, she […] started to rotate her probe counterclockwise. The mid‐portion of the bony roof is bent, causing the femoral head to disappear completely from the image. (ID1)

Another nurse had limited knowledge of SP criteria. Consequently, after looking at the hip joint image on the screen, she mistakenly judged that the SP had been captured based on incorrect criteria.

#### 5.4.6. Infant Not in a Straight Lateral Position

Nurses were required to place the participants in a side‐lying position to direct the ultrasound onto the hip joint. However, infants moved due to crying, preferred body position, or an arched back, which could lead to a loss of proper alignment. Nurses learned to position themselves and the caregiver in front of the infant’s abdomen and back, using rolled towels in front of each knee to keep the infant still. However, if nurses and caregivers insufficiently held the participant down with their hands or knees, their body would tilt away from the vertical–lateral position. Consequently, it was difficult to visualize the femoral head or SP.

The caregiver’s knees were not in contact with the towel placed on the infant’s abdominal side, causing the infant to lean slightly forward. ID1 did not place their knees against the towel on the infant’s back. […] The pediatric orthopedist (author) told ID1, “The femoral head does not appear round, which feels off.” (ID1).

However, a nurse who objectively assessed the participant’s position could reposition the image vertically and display it. The nurse captured the SP by swiftly placing the probe vertically when the infant’s movement was briefly paused and the caregiver repositioned their hold on the infant.

The caregiver grasped the infant’s raised leg with their right hand and repositioned it so that the toes of the right foot touched the back of the left knee. […] As the infant’s movement temporarily ceased and ID1 continued to focus on the tablet, she rotated the probe with both hands a few degrees to the right and completed capturing the SP. (ID1).

#### 5.4.7. Failure to Improve the Procedure

During the ultrasound training program, the nurses received advice from the authors regarding anatomical knowledge and ultrasound examination methods using the probe. However, there were times when the nurse became so focused on the examination that they did not respond to the advice. One nurse was instructed to make a broad sliding movement with the probe but was unable to follow the instructions or locate the femoral head.

The author told ID3, “Rather than moving it in small adjustments when the femoral head is not found, move the probe more broadly until you find it…” However, ID3 once again began sliding the probe back and forth in small movements of less than 1 cm. (ID3).

## 6. Discussion

This study is the first to systematically identify and categorize the challenges that nurses face in learning ultrasound techniques using the Graf‐method [16]. The results revealed four distinct challenge domains: (1) knowledge‐related, (2) skill‐related, (3) challenges involving both knowledge and skills, and (4) attitude‐related. Unlike previous research that focused primarily on clinically based nurses, who could serve as physician substitutes [16], this study targeted municipal PHNs engaged in in‐home visits. In ultrasound training programs, knowledge is reinforced through e‐learning, and knowledge and skills are enhanced through hands‐on seminars [23]. Understanding the challenges nurses face in advance may help educators improve their teaching methods to enable nurses to perform high‐quality and safe ultrasound examinations. Municipal nurses have only recently begun DDH ultrasound training [23, 24], and currently, only a few municipalities offer such programs [24, 33], resulting in a limited number of nurse cohorts practicing this skill. However, the three nurses included in this study represented a wide range of ages and educational backgrounds and included individuals without prior experience in the most common DDH screening training. This study represents an early and innovative effort that highlights the challenges PHNs may encounter in the future when learning ultrasound skills.

First, this study revealed that a lack of anatomical knowledge, particularly regarding the appearance of structures on ultrasound images and the SP, is a key factor hindering the accurate identification of the femoral head and SP. Despite receiving education on ultrasound imaging and hip anatomy through e‐learning and seminars [23], the targeted PHNs did not achieve sufficient knowledge retention. This contrasts with previous studies, in which approximately 90% of hospital nurses newly trained using the Graf‐method could interpret images accurately and successfully capture SP [16]. Previous studies focused on hospital nurses who could serve as substitutes for physicians because of physician shortages [16], whereas this study targeted municipal PHNs. Japanese PHNs working with infants focus more on social and daily health determinants than anatomical knowledge [34]. This finding suggests that traditional lecture‐based learning alone is insufficient for nurses unfamiliar with anatomical imaging. One possible approach is using three‐dimensional anatomical models that allow nurses to correlate bone structures with ultrasound images in real time [35]. Teaching the three‐dimensional structure of the hip joint using bone models, along with its correlation to two‐dimensional ultrasound images, may help nurses deepen the anatomical knowledge necessary for ultrasound examinations they have not yet fully mastered. Additionally, incorporating discussions of imaged case data into training and promoting interpretation that integrates anatomy with ultrasound findings may improve the retention of practical knowledge.

Second, this study identified the challenges faced by nurses in estimating the femoral head position, moving the probe correctly, and setting up equipment. These challenges stem from technical knowledge gaps and a lack of sensor‐motor experience, such as detecting the greater trochanter and linking probe movements to the screen. While verbal‐ and video‐based training provides theoretical guidance [23], these methods do not effectively translate into hands‐on proficiency. The Graf‐method has not thoroughly examined the delays caused by these factors [10], and the current training does not adequately develop the sensorimotor skills necessary for palpating anatomical landmarks, such as the greater trochanter. One critical issue was that the phantom dolls used in the training lacked realistic palpability, making it difficult for nurses to develop tactile sensitivity for detecting the femoral head. Addressing this limitation may involve formalizing instruction on the sensory experience of femoral head palpation and offering hands‐on training that incorporates tactile learning methods, such as haptic feedback simulators designed to replicate an infant hip joint. Live, supervised practice with real infants under expert guidance may enhance skill acquisition. Furthermore, the Graf‐method requires rotating the tablet screen 90° from the infant’s actual position [36, 37], likely causing misalignment between visual and proprioceptive information and making probe‐screen coordination more challenging. To address this, structured task decomposition training may be beneficial, as nurses first master the basic probe movements before integrating full imaging techniques. An immediate solution for integrating this technique could be to add arrow marks to the tablet to indicate the correspondence between the direction of probe movement and image orientation.

Third, this study revealed that the nurses’ posture, probe handling, and infant position interfered with the probe’s vertical placement, hindering the capturing of the SP. Previous studies on cardiac and hip ultrasonography have highlighted similar challenges in probe stability and positioning [16, 38]. However, no prior research has thoroughly examined the specific impact of the home settings on imaging outcomes, making this study the first to highlight these unique constraints. Despite using e‐learning and hands‐on seminars [23], these methods do not adequately address the challenges nurses face in probe handling and positioning. The Graf‐method recommends using specialized equipment, such as a cradle and probe stabilizer, to secure both the probe and the infant [5, 37]. However, conducting ultrasound examinations in home settings without access to such devices likely contributed to nurse and infant movement, affecting imaging success [13]. Seeking assistance from caregivers is essential [37] because a caregiver’s manual fixation is considered as effective as Graf’s fixation device used in medical settings [12]. Additional solutions, such as an alert system to notify when the probe is tilted and a portable fixation device to maintain the infant’s lateral position, may be necessary. Additionally, difficulties with probe handling were more evident among older nurses, suggesting an age‐related decline in hand–eye coordination [39]. Reducing probe weight and increasing fine motor skills training may help improve these techniques.

### 6.1. Strengths and Limitations

This study was the first to systematically identify and categorize nurses’ challenges in learning ultrasound techniques using the Graf‐method in in‐home settings [16]. Understanding these challenges can enhance education on ultrasound hip‐screening techniques and strengthen nurse‐led ultrasound hip‐screening initiatives for the early detection of hip dysplasia in home settings. Despite these strengths, this study has certain limitations. First, the development of municipal nurses in Japan responsible for conducting DDH ultrasound examinations is still at an early stage, with both the overall study population and the number of failure cases being limited. Furthermore, the participants may have been highly motivated early adopters with inherently higher potential, and their performance might have exceeded that of nurses in municipalities that have not yet implemented the program. These factors limit the generalizability of the findings. Second, challenges were identified through video recordings, which did not allow for triangulation or exploration of the psychological factors that influence nurses’ actions. Third, challenges were hypothesized based on previous research and discussions, making it unclear whether they were directly related to the acquisition of ultrasound techniques. However, given that the nurses were novices in ultrasound techniques, it is reasonable to assume that many of the identified challenges were related to acquiring these skills. Fourth, only one camera was used to minimize the psychological burden on the nurses, limiting the scope of the observed challenges and their underlying causes.

### 6.2. Recommendations for Further Research

Future studies should develop effective and efficient educational programs that address the challenges identified in image acquisition to improve the success rate of nurse‐led ultrasound screening. Integrating hip anatomy, imaging interpretation, and proper probe orientation into training is crucial for enhancing nurses’ competencies. Identifying the psychological pressures experienced by nurses would further support the findings of this study and contribute to the development of an educational program that boosts nurses’ confidence. Additionally, future research should examine the association between hands‐on training performance and imaging success rates during home visits to enhance nurse‐led ultrasound hip screening globally.

### 6.3. Implications for Policy and Practice

Improving health disparities and promoting healthy child development remain global priorities [40]. Nurse‐led home visit programs improve maternal and child health [41, 42]. Home visits provide excellent opportunities for nurses to observe the hip joints of all newborns and infants [24], especially in areas with limited access to medical institutions. Although physical examination alone has limitations in detecting DDH, early and accurate detection through ultrasound and subsequent preventive interventions is feasible [4, 8]. Therefore, nurse‐performed universal ultrasound during home visits is important for reducing DDH‐related health disparities and promoting lifelong healthy development [16, 23].

Additionally, nurse‐performed universal ultrasound during home visits also affects healthcare costs. To date, the cost‐effectiveness of hip screening has been evaluated primarily based on examinations performed by physicians [6, 7]. However, integrating ultrasound screening into existing healthcare frameworks by training nurses to perform these examinations could significantly reduce costs and expand screening coverage to include more children than physician‐led screening. Early detection of DDH may reduce surgery rates, complications, and healthcare costs [5]. DDH leads to hip osteoarthritis in the future [43]. Early detection and treatment of DDH may also help reduce future medical and long‐term care costs. The national government should support the education of nurses and trainers to help reduce overall healthcare costs.

A shift in educational policy should be considered when integrating ultrasound hip‐screening training with basic nursing education. Despite evidence that physical examinations have poor sensitivity [4] and that ultrasound screening provides a more reliable assessment of hip joint conditions [10], failure to incorporate this training into nursing education may compromise patient benefits and violate nursing ethics. Currently, learning about ultrasound hip screening is not a mandatory part of the nursing school curricula, and the three nurses in this study had no prior education in either DDH or ultrasound. Introducing ultrasound training at an earlier stage, while students are still developing hand–eye coordination, could facilitate the acquisition of anatomical knowledge and ultrasound techniques. This approach may allow nurses to perform hip ultrasound screenings immediately after entering the workforce and may contribute to the early detection of DDH in community settings.

## 7. Conclusion

This study highlights the challenges nurses face when learning ultrasound techniques using the Graf‐method, particularly in home settings. The main challenges include insufficient anatomical knowledge, difficulty estimating the femoral head position, challenges in probe handling and infant positioning, and sensory skill deficits related to ultrasound equipment. We emphasize the importance of providing training programs that incorporate three‐dimensional anatomical understanding, enhance sensory experiences, and improve probe‐screen coordination. Additionally, addressing the challenges related to posture, equipment handling, and fine motor skills can improve ultrasound proficiency. To bridge the gap between knowledge and practice, further refinement of the educational program would improve nurses’ competencies and increase the accuracy of ultrasound hip screening. This advancement promotes high‐quality and safe early detection of DDH within the home setting, leading to preventive interventions by nurses and infants’ caregivers.

## Funding

This research was supported by the Ministry of Education, Culture, Sports, Science and Technology as “Developing a Research Data Ecosystem for the Promotion of Data‐Driven Science” (Grant number: none, to Kyoko Yoshioka‐Maeda) and the JSPS KAKENHI grant as “Development of educational program and implementation of ultrasound hip screening during newborn/infant home visits” (Grant number: 24K02762, to Kyoko Yoshioka‐Maeda) and “Development of a real‐time assessment method for hip dislocation using a flexible probe” (Grant number: 24K22226, to Kyoko Yoshioka‐Maeda).

## Conflicts of Interest

The authors declare no conflicts of interest.

## Supporting Information

SRQR guideline: We followed the SRQR guideline for reporting qualitative research.

## Supporting information


**Supporting Information** Additional supporting information can be found online in the Supporting Information section.

## Data Availability

The data are not publicly available due to privacy or ethical restrictions.
