# Supplementary figures and images for: Nurses’ Challenges in Performing Ultrasound Hip‐Screening Techniques for Home Visits to Newborns and Infants: A Descriptive Case Study
Source: J Nurs Manag. 2026 Apr 13;2026:8226375. doi: 10.1155/jonm/8226375 (PMC13072062; doi:10.1155/jonm/8226375)

## Slide 1
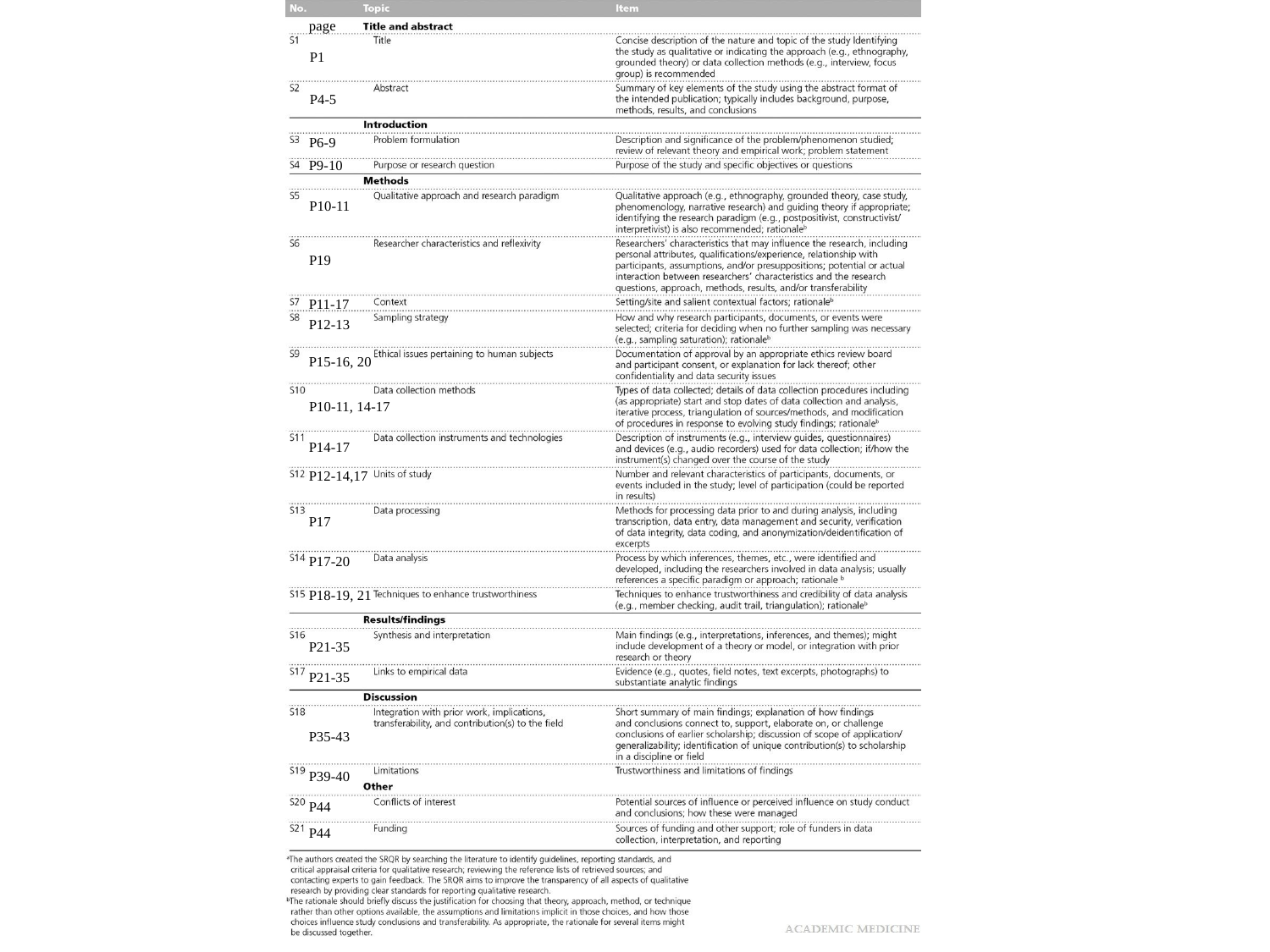

page
P1
P4-5
P6-9
P9-10
P10-11
P19
P11-17
P12-13
P15-16, 20
P10-11, 14-17
P14-17
P12-14,17
P17
P17-20
P18-19, 21
P21-35
P21-35
P35-43
P39-40
P44
P44

Supplement: Supplementary file 1 — Supporting Information Additional supporting information can be found online in the Supporting Information section. [file JONM-2026-8226375-s001.pptx]
